# Author Correction: Determining protein structures in cellular lamella at pseudo-atomic resolution by GisSPA

**DOI:** 10.1038/s41467-024-47528-6

**Published:** 2024-04-23

**Authors:** Jing Cheng, Tong Liu, Xin You, Fa Zhang, Sen-Fang Sui, Xiaohua Wan, Xinzheng Zhang

**Affiliations:** 1 National Laboratory of Biomacromolecules, CAS Center for Excellence in Biomacromolecules, Institute of Biophysics, Chinese Academy of Sciences, Beijing, 100101 China; 2 High Performance Computer Research Center, Institute of Computing Technology, Chinese Academy of Sciences, Beijing, 100190 China; 3 State Key Laboratory of Membrane Biology, Beijing Advanced Innovation Center for Structural Biology, School of Life Sciences, Tsinghua University, Beijing, 100084 China; 4 Beijing Institute of Technology, Beijing, 100081 China

**Keywords:** Cryoelectron microscopy, Cryoelectron tomography, Software, Data processing

Correction to: *Nature Communications* 10.1038/s41467-023-36175-y, published online 15 March 2023

The original version of this Article contained an error in “Introduction”, which incorrectly read ‘We examine the feasibility of applying GisSPA to FIB-thinned *Pennisetum purpureum* (*P. purpureum*) cellular lamellae.’ The correct version states ‘*Porphyridium’* in place of ‘*Pennisetum’*. This has been corrected in both the PDF and HTML versions of the Article.

