# Supplementary material for: Determining protein structures in cellular lamella at pseudo-atomic resolution by GisSPA
Source: Nat Commun. 2023 Mar 15;14:1282. doi: 10.1038/s41467-023-36175-y (PMC10017804; doi:10.1038/s41467-023-36175-y)
Supplement: Supplementary file 1 — Supplementary Information [file 41467_2023_36175_MOESM1_ESM.pdf]

Supplementary Figures

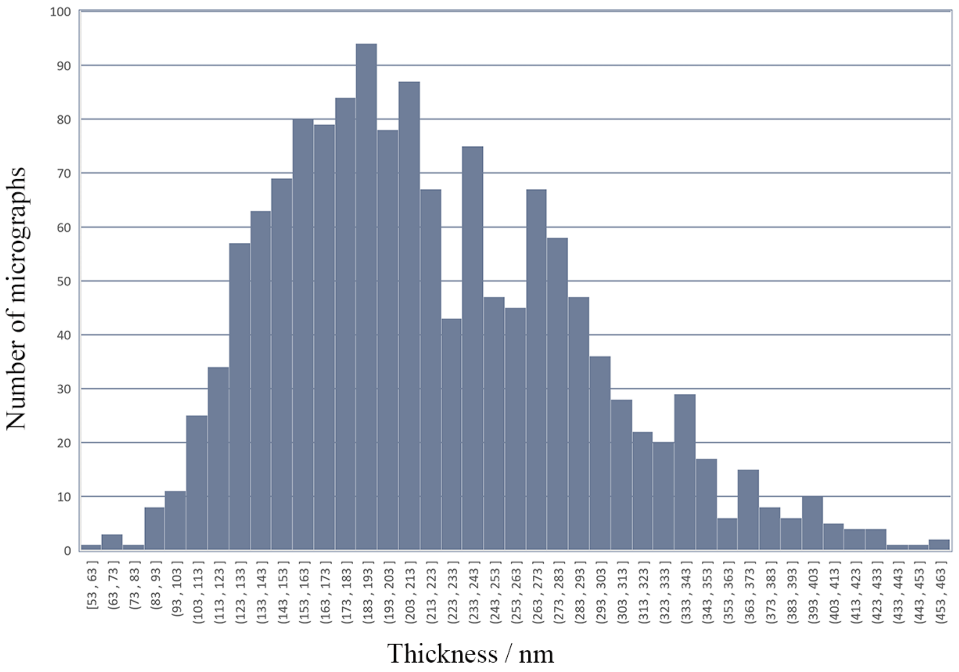

Supplementary Fig. 1. Histogram of estimated thickness distribution of *P. purpureum* lamellae. Thickness of the 1,437 micrographs of *P. purpureum* lamellae ranges from 60 to 460 nm. 95% of the lamellae are 100–350 nm thick and 50% of the lamellae are thicker than 200 nm. Source data are provided as a Source Data file.

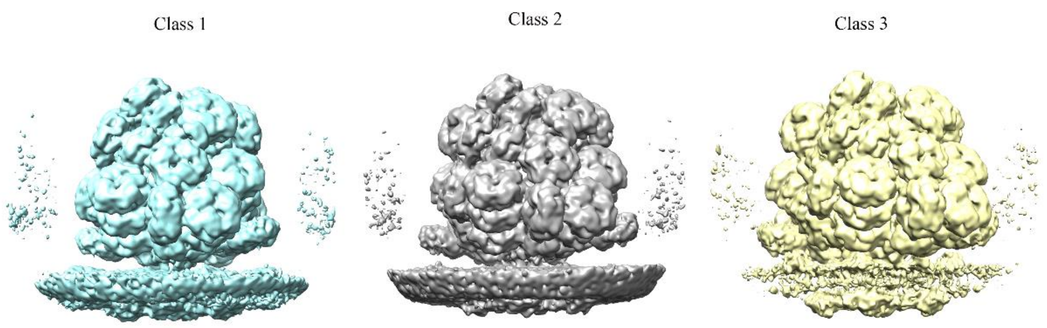

Supplementary Fig. 2. 3D classes of PBS particles detected by GisSPA. Each class contains thylakoid membranes and weak PBS densities nearby.

a

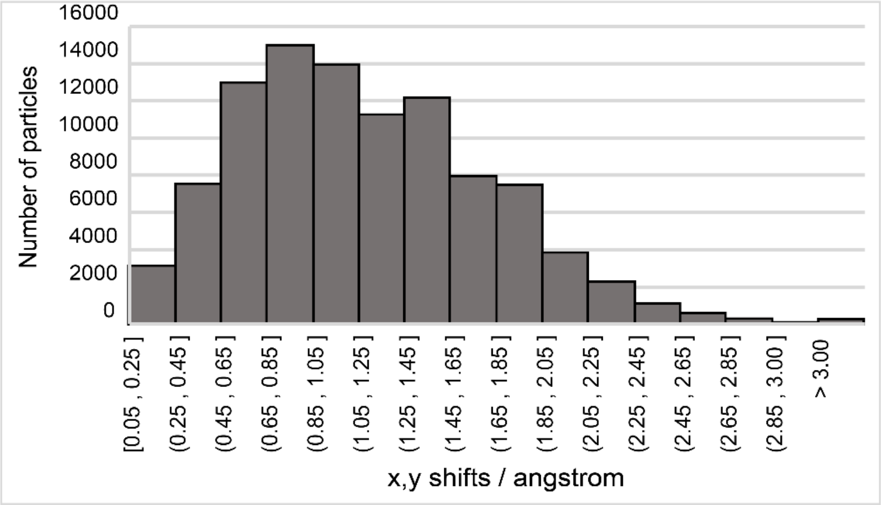

b

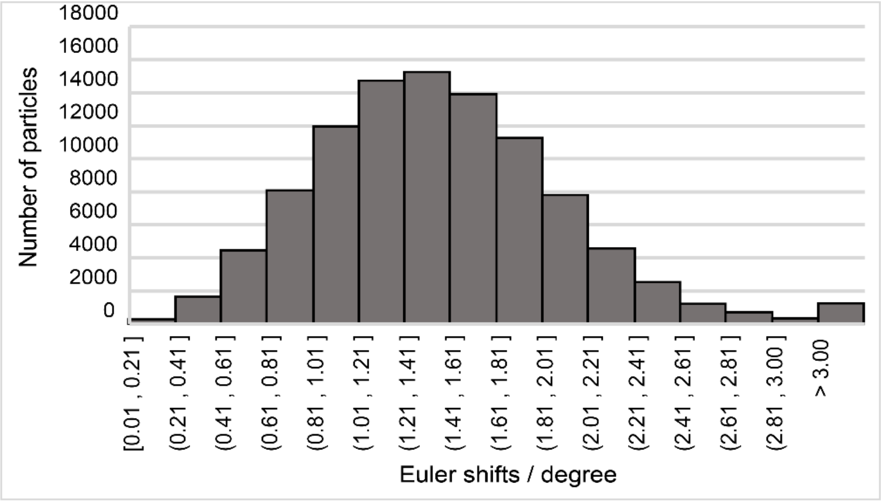

Supplementary Fig. 3. Displacement of PBS particles. a Statistics on the displacement of PBS particle locations. b Statistics on the displacement of PBS particle orientations. Source data are provided as a Source Data file.

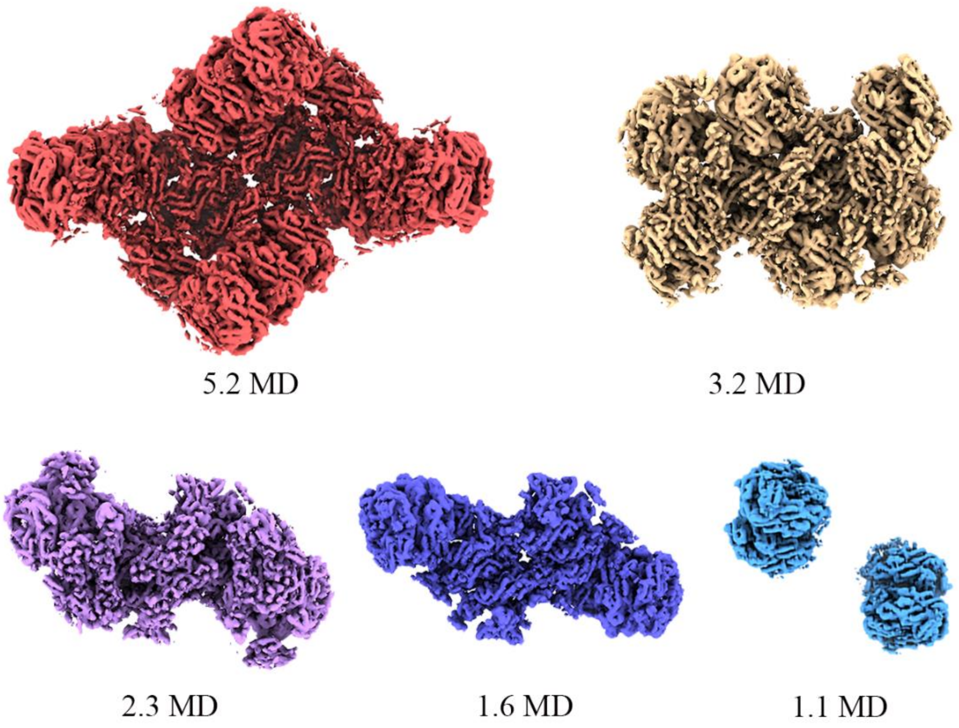

Supplementary Fig. 4. 3D maps used as models in different molecular weights. Maps are segmented from the PBS map with a molecular weight of 5.2 MD (red), 3.2 MD (orange) , 2.3 MD (light purple), 1.6 MD (dark purple) and 1.1 MD (blue) respectively. All maps have a 2-fold symmetry.

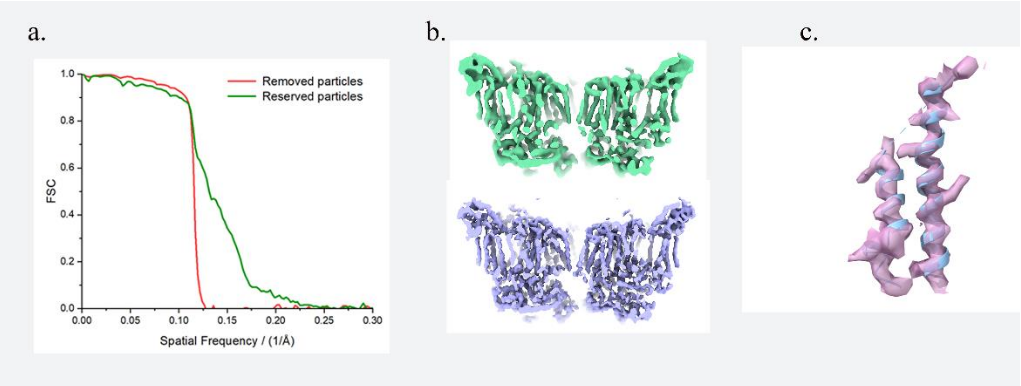

Supplementary Fig. 5. Processing on PSII complex. a FSC curves of reconstructions from removed particles (red) and reserved particles (green) distinguished by sorting. b Densities of map at 5.7 Å before sorting (green) and densities of map after sorting at 4.7 Å (purple). c Densities of α-helices from the 3.9-Å map, the map was fitted with PDB (6jio). Source data are provided as a Source Data file.

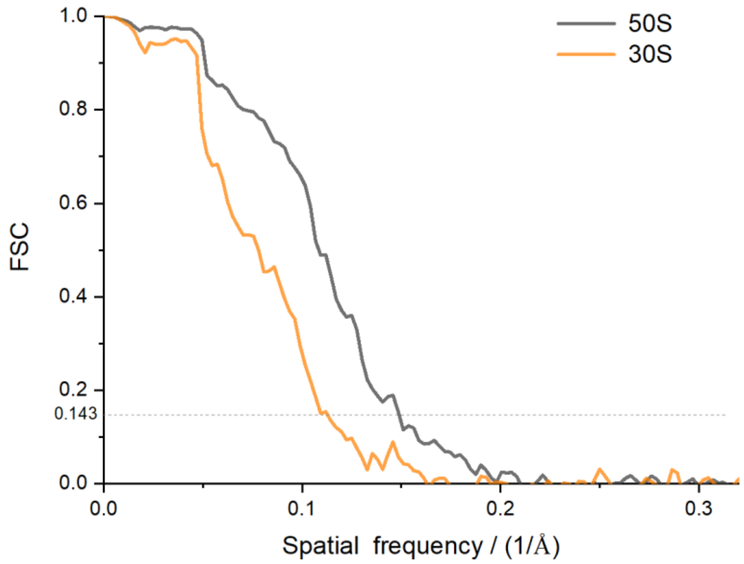

Supplementary Fig. 6. FSC of ribosome. Standard FSC curves of 50S subunit (gray) and 30S subunit (orange). Source data are provided as a Source Data file.

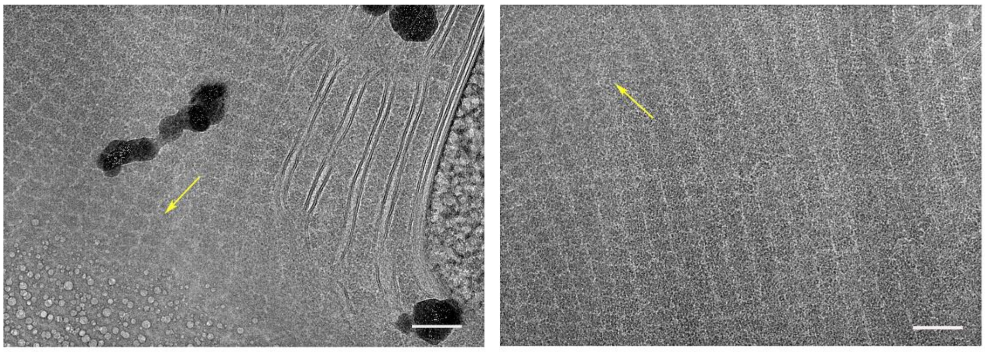

Supplementary Fig. 7. Micrographs contain double illuminated area. Areas with double exposures are indicated by yellow arrows, scale bars represent 100 nm.

Supplementary Table 1. **Cryo-EM data collection, refinement statistics.**

	PBS(GisSPA)				PSII (GisSPA)
	PBS	PSII-1	PSII-2	PSII-3	PSII
**Data collection and processing**					
Magnification	53,000	53,000	53,000	53,000	53,000
Voltage (kV)	300	300	300	300	300
Electron exposure (e–/Å^2)	35	35	35	35	35
Defocus range (μm)	-1.0~-6.0	-1.0~-6.0	-1.0~-6.0	-1.0~-6.0	-1.0~-6.0
Pixel size (Å)	1.668	1.668	1.668	1.668	1.632
Symmetry imposed	C1	C1	C1	C1	C2
Initial particle images (no.)	651,784	651,784	651,784	651,784	482,888
Final particle images (no.)	502,916	229,758	229,758	147,568	23,410
Map resolution (Å)	3.4	3.6	3.7	4.4	3.9
FSC threshold	0.143	0.143	0.143	0.143	0.143
Map resolution range (Å)	3.4-8				
